# Gamification in Diplomacy Studies as an Effective Tool for Knowledge Transfer: Questionnaire Study

**DOI:** 10.2196/32996

**Published:** 2022-04-25

**Authors:** Mihai Ovidiu Cercel

**Affiliations:** 1 Department of International Relations and European Integration National University of Political Studies and Public Administration Bucharest Romania

**Keywords:** modern diplomacy, international relations, innovations in learning experience, gamification, serious games, role play design, knowledge transfer, competency development

## Abstract

**Background:**

Graduate education in modern diplomacy poses several challenges, as it requires several competencies to be developed before diplomatic service is joined. Incorporation of simulation games can have a positive impact on the design of international relations and diplomacy learning process. We have designed a novel role play game (MAEDRI) to simulate part of the activities of a typical Ministry of Foreign Affairs.

**Objective:**

This study aims to evaluate the effectiveness of MAEDRI in transferring knowledge in international relations education programs at the National University of Political Studies and Public Administration, Bucharest, Romania, across a 4-year period.

**Methods:**

The game enrolled master’s level graduate students. The data were collected through a voluntary and anonymous questionnaire between 2017 and 2020. At the end of each of the 4 editions we organized debriefing sessions that gave students the opportunity to provide feedback on their experience with this exercise, level of collaboration within the team, lessons learned, and to make suggestions for improvements. Using an online questionnaire, we measured the participants’ perception regarding the level of effectiveness in increasing knowledge transfer, motivation, and engagement. Questionnaire data were consolidated in percentages for each item.

**Results:**

A total of 49 participants completed the study. A total of 24 skills (13 professional and 11 social skills) were assessed. We identified a strong positive correlation between stress management and conflict management (*r*=.86; *P*<.001) as well as significantly positive correlations between building relations within the team and the ability to dialog and be persuasive (*r*=.7; *P*<.001), between procedure compliance and planning and organizing the work (*r*=.69; *P*<.001), and between analysis capacity and decision based on data received (*r*=.68; *P*<.001). Among social skills, self-control, confidence, and flexibility were the most substantially improved.

**Conclusions:**

We describe several benefits of a novel game, used as an education tool to enhance a series of competencies necessary in international relations studies. Our results demonstrate a significant level of student engagement and motivation while playing MAEDRI, improvement of several essential skills, and enhanced knowledge transfer to real-life situations. While the data are encouraging, further research is needed to evaluate the full impact of role play as an effective experiential learning method.

## Introduction

International relations and diplomacy are in constant transformation, mostly as a result of the necessary adaptation to the digital revolution that has led to the emergence of new platforms that cover events in real time, while enabling easy access to global communication. Innovations in teaching international relations are critical for the success of graduate programs in these fields, which should remain adaptable and reflective of the new, constantly evolving trends.

The fundamental mission of education is to transfer knowledge in various fields and to assess the competencies acquired, by measuring the ability of students to use in practice what they learnt theoretically [1,2]. Diplomatic skills are difficult to acquire exclusively from theoretical information. In addition, practicing diplomacy is almost impossible without being a career diplomat, given that diplomatic activities are largely based on confidential information. Therefore, one of the challenges when creating successful graduate programs in diplomacy and international negotiations consists of providing learning experiences that are as close to real-life scenarios as possible. To meet some of these challenges, we designed and implemented an innovative simulation game (SG).

SGs combine the features of serious games and simulation and have emerged as powerful tools to improve learning outcomes, by facilitating a better understanding of specific issues, via simulated experience [3-6]. Garris et al [7] argued that both serious game design and the gamification of learning increase learning outcomes, either directly (games) or indirectly through an alteration of contextual learner behavior. Vlachopoulos and Makri [8] see the usage of serious games in higher education as a necessary step forward in pedagogy toward a student-centered environment. Moreover, gamification has the potential to enhance motivation and induce behavioral changes, while fostering team work and promoting friendly competition in different work contexts [9-11].

Using role play learning and personalized learning to support the development of different competencies is not a new educational method, but it is a topic often described in recent years as a powerful tool to enhance learning by challenging students’ creativity [12-15]. Dynamic grouping strategies are effective in enhancing students’ learning [16,17].

A plethora of research studies have supported the use of SGs as a way to increase motivation, engagement, and learning outcomes [4,5,7,18-24] by enhancing attention, active learning, feedback, and consolidation, the 4 main pillars of learning [5,23].

The class curriculum in international relations courses includes, almost without exception, different diplomatic simulations. Published evidence from studies on experiential learning literature points to several impactful learning aspects of simulations, such as setting up objectives, creating opportunities for interactions, or enhancing teaching notes, but there is little research regarding the students’ perception of relevant skill development [8,13,16,19]. As young adults, students are often engaged in cyclical, experiential learning, as described by Kolb’s Learning Cycle [25]. As discussed by Duffy [26], individuals differ in their learning process, how they process information, and how they assimilate and use it in future actions. Kim [27] thinks that game theory, together with other behavioral disciplines, may offer a better understanding of political science concepts.

When teaching political science, especially diplomacy, one is confronted with a major challenge—how to develop relevant, applicable skills, taking into consideration the fact that in real-life situations (occurring, for example, in ministries of foreign affairs [MFAs]) there is a tangible need for access to confidential information. The benefits of using a mix of personalized learning and role playing in political and social sciences are well demonstrated in pedagogical literature [27-31]. Hardy and Totman [32] consider that the use of such mixed approaches in international relations requires discipline-specific examination. Analyses of international affairs and case studies are the main tools in the teacher’s toolbox. Online diplomatic role play or simulations of debates in International Organizations (such as Model United Nations [UN] or the North Atlantic Treaty Organization [NATO] Conference model) are the most common simulations in all universities.

Nevertheless, such simulations are often inadequate because either they focus on specific situations from the past (and the students try to adopt the same, obsolete approach) or they use imaginary situations, designed to promote an understanding of the internal mechanisms of each diplomatic organization [33-35]. The question is whether role play simulations in international relations (online or face-to-face exercises) generate measurable and meaningful outcomes in developing specific skills [36-38]. Gamification helps students to better understand how theoretical concepts could be applied for solving real-life problems, and whether those skills are retained longer than classical learning methods [39,40].

To increase the impact of graduate-level education on diplomacy, we developed MAEDRI, an innovative SG that simulates parts of the diplomatic activity typically seen in international relations. We hypothesized that the application of game elements in learning diplomacy will enhance knowledge transfer. In addition, we hypothesized that game participants would develop or enhance social skills necessary for practicing international relations and be better prepared for their professional life. Between 2017 and 2020, a total of 150 master’s level graduate students have played MAEDRI, which was administered as part of the Diplomacy and International Negotiation Master’s Program at the National University of Political Studies and Public Administration, Bucharest, Romania. All student participants were, at the time of this study, enrolled in Master’s Programs such as International Relations and European Integration, Conflict Analysis, Diplomacy and International Negotiation, and Security and Diplomacy. The study ran in 4 consecutive annual editions. Our aim was to evaluate the effectiveness of experiential learning through gamification, and to assess the impact of MAEDRI on student preparedness and their ability to develop specific capabilities, typically used by diplomats in their professional life. Ultimately, this study examines how gamification can boost students’ competencies in international relations, improve their ability to apply theoretical concepts to practice, motivate them, and enhance their engagement during the educational process.

## Methods

### Overview

We developed MAEDRI, a new role play game that explores the interactions between real policy, politics, and students’ capabilities. The game is primarily designed for participants who are studying international relations or political sciences, at graduate (master’s) level. The MAEDRI role play has been organized annually since 2016.

### Study Participants

All study participants are graduate students enrolled in master’s level programs in International Relations, Diplomacy and International Negotiations, Conflict Analysis, and Security and Diplomacy at the National University of Political Studies and Public Administration, Bucharest, Romania.

All participants provided written consent to participate in the research. A total of 150 students who have played MAEDRI received a message containing information about the study via the MAEDRI Facebook group, which was restricted to students only. The participants were not chosen randomly, and participation in the research was voluntary.

### Ethical Considerations

Per institutional guidelines on survey studies, the Department of International Relations and European Integration within the National University of Political Studies and Public Administration in Bucharest, Romania, deemed that this study met criteria for exemption from review by the Quality Assurance Committee.

### Study Design

This study evaluated students’ perceptions of effectiveness after playing MAEDRI. The data were collected through an anonymous questionnaire consisting of 9 matrix grid questions. The questionnaire was administered online, through the SurveyMonkey platform [41]. Research participants had to meet the inclusion criterion (ie, participation in one of the MAEDRI annual editions between 2017 and 2020). The questionnaire sought feedback on the participants’ perception regarding their level on several key learning skills, and the extent the acquired competencies were put in practice in real life during their university studies and within their current work environment. In Romania, most graduate students are employed, and work during their studies. To determine whether the knowledge was transferred effectively, students were invited to self-evaluate on a range of professional and social competencies, before and after the role play simulation.

The questionnaire included response options along a 5-point Likert scale, ranging from 1 (strongly disagree) to 5 (strongly agree). The online survey was conducted in accordance with the Checklist for Reporting Results of Internet E-Surveys (CHERRIES) checklist [42]. The average time to complete the questionnaire was 15 minutes.

### Statistical Methods

Questionnaire data were consolidated in percentages for each item and analyzed using the MS Excel (Microsoft) data analysis module.

*P*-value was determined to validate the results of statistical analysis. A *P*-value <.001 was considered significant. Because of the exploratory nature of this study, we used the Cronbach α value. A high value for Cronbach α (<.8) indicates a good consistency of the items in the scale and helps validate the reliability of the questionnaire (Table 1). A 1-sample 2-tailed *t* test against the neutral value in the 5-point Likert rating was used to assess the responses to the 24 evaluated skills (Textbox 1).

**Table 1:** Normality and scale reliability results.

Skills	*P*-value	Cronbach α	*t* statistic (*df*)
Professional	<.001	.909	8.63 (48)
Social	<.001	.871	5.71 (48)

**Transferable skills through the MAEDRI game.**

**Professional skills**

- Searching and filtering information in virtual space
- Problem solving
- Task partitioning
- Assertiveness
- Attention to details
- Giving and receiving feedback
- Decision making based on data received
- Analysis capacity
- Change management
- Planning and organizing the work
- Writing reports using a diplomatic language
- Fluency and concision
- Procedures compliance

**Social skills**

- Ability to dialog and be persuasive
- Building relations within the team
- Team coordination
- Self-control and confidence
- Flexibility
- Team motivation
- Initiative and creativity
- Care for order, quality of work, and accuracy
- Emotional intelligence
- Conflict management
- Stress management

## Results

### MAEDRI Game Design and Implementation

**Figure 1:**
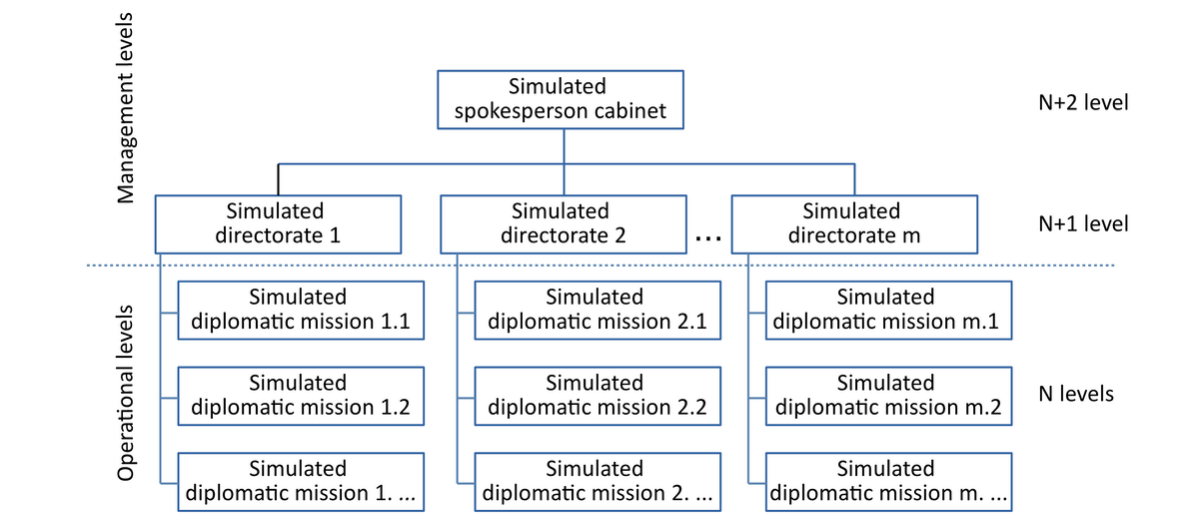
The organizational chart of the simulated organization - MAEDRI.

The preparation of the exercise includes advertising and promoting the game, selecting the “future diplomats” for simulated diplomatic missions, allocating the roles based on their interests and knowledge of different foreign languages, and promoting the activities on social media (Multimedia Appendix 1). The more senior, final-year students assume management roles at simulated headquarters. A system of communication and a chain of command are established to send information and receive feedback (Figure 1). Once roles are assigned, participants will conduct their research online, to identify all the relevant local media in their “host country” and select the most important media websites to generate a balanced portfolio. Google Translate or similar online tools were employed for translation to Romanian.

Every day during the role play game, the students in simulated diplomatic missions (N level) were asked to monitor all official media webpages selected during the preparation period in the “host” country (Figure 1). They also analyzed, selected, and filtered the information, focusing on several criteria, such as the specifics of bilateral relations between Romania and the country where the simulated diplomatic mission was based; the political, economic, social, and cultural environment in the “allocated” country; and the positions expressed by the representatives of the allocated country regarding the international topics. The participants were required to provide a daily written report (with a limit of 400 words per article) by observing the diplomatic style in terms of language and concision.

The resulting report was subsequently sent to the next decision level—the simulated directorates—organized on geographical or functional criteria by grouping 4-7 simulated diplomatic missions. “Late” reports, that is, those submitted after the agreed upon deadline, were generally not considered. Exceptions were made only for unexpected events occurring in the “simulated” country.

The chain of command simulates the various divisions of an ordinary MFA. In the MAEDRI game (Figure 1), we simulate only 2 management levels: simulated directorates (N+1 level) and simulated spokesperson cabinet (N+ 2 levels). For the chain of command, the tasks focused on analyzing and comparing information obtained from various sources, filtering reports based on the setup criteria, studying the allocated geographic space, and compiling the selected information using a diplomatic language, to finalize the report in a format that is relevant and interesting to the public. The decisions were made collectively in groups of 7-9 students, similar to a newspaper editorial office.

The simulated spokesperson cabinet is the last and the highest hierarchical structure in the simulated MFA. This structure is responsible for selecting the most relevant articles received from the general directorates and publishing them on the MAEDRI simulated MFA Facebook page (Multimedia Appendix 2, in Romanian only).

At the end of each edition debriefing sessions were organized that gave students the opportunity to provide feedback on their experience with this exercise, level of collaboration within the team, lessons learned, and to make suggestions for improvements.

The skills acquired during role play can be separated into 2 categories: professional and social (Textbox 1).

Approximately 10% (5/49) of the respondents participated in 2 successive editions of the role play game (first time as lower-level “diplomats” in the simulated diplomatic missions and second time as “diplomats” in a managerial position), but the online platform used for data collection allowed a single questionnaire to be completed from the same IP address.

### Characteristics of Study Participants

The study involved 49 respondents out of 150 students who participated in the MAEDRI simulations. The demographic characteristics of the 49 participants are representative of the entire group of 150 in terms of sex, age, and work status. Table 2 presents their demographic features, their participation in MAEDRI simulations, and employment situation.

**Table 2:** Descriptive characteristics of participants (n=49).

Characteristics		Value
**Sex, n (%)**		
	Male	11 (22)
	Female	38 (78)
**Age (years), n (%)**		
	20-24	17 (35)
	25-30	24 (49)
	Over 30	6 (12)
	Unknown	2 (4)
**Participant in, n (%)**		
	Edition 2017	11 (22)
	Edition 2018	8 (16)
	Edition 2019	9 (18)
	Edition 2020	21 (43)
**Employment situation, n (%)**		
	Working in the international relations field	17 (35)
	Working in other sectors	20 (41)
	Unemployed	11 (22)
	Unknown employment status	1 (2)

### Correlations of Measurements

A strong positive correlation (Table 3 and Multimedia Appendix 3) was found between stress management and conflict management (*r*=.86; *P*<.001) as well as significantly positive correlations between building relations within the team and the ability to dialog and be persuasive (*r*=.7; *P*<.001), between procedures compliance and planning and organizing the work (*r*=.69; *P*<.001), and between analysis capacity and decision based on data received (*r*=.68; *P*<.001). No negative correlations were found.

**Table 3:** Most significant correlations.

Skills	Decision making	Planning and organizing the work	Ability to dialog and be persuasive	Conflict management
Analysis capacity	0.6851	0.3702	0.3794	0.5543
Procedures compliance	0.2638	0.6908	0.3523	0.2237
Building relations within the team	0.4338	0.3283	0.7016	0.2959
Stress management	0.5739	0.4708	0.3339	0.8568

### The MAEDRI Simulation Game Enables Knowledge Transfer and Improves Professional Competencies Diplomacy Studies

**Figure 2:**
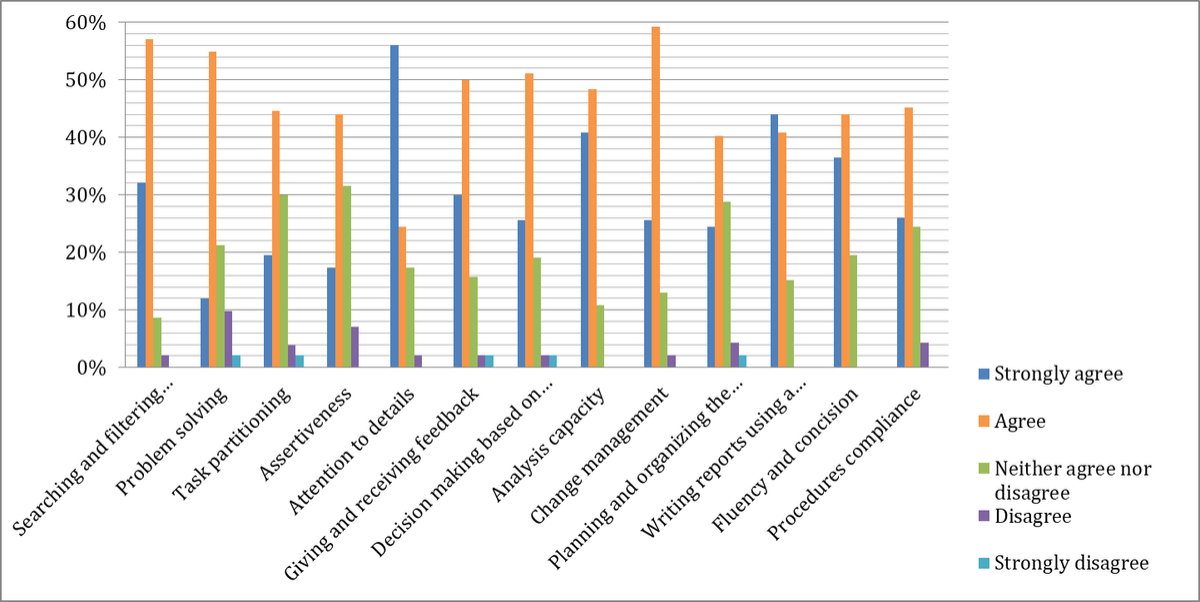
Improvement of professional competencies after MAEDRI game.

Most study participants either strongly agreed or agreed that the role play helped them in developing professional skills in the field of international relations (Figure 2). Approximately 39% (19/49) of participants either strongly agreed or agreed that the simulation helped them to better understand the theoretical concepts and to put in practice what they learned. The most remarkable results are observed for searching and filtering information in virtual space, where 88% (43/49) of participants strongly agreed and agreed that the role play simulation improved their competencies. The same tendency for attention to details was observed, where 73% (36/49) of participants strongly agreed and agreed.

### The MAEDRI Game Enhances Social Skills Necessary for a Professional Career in International Relations

**Figure 3:**
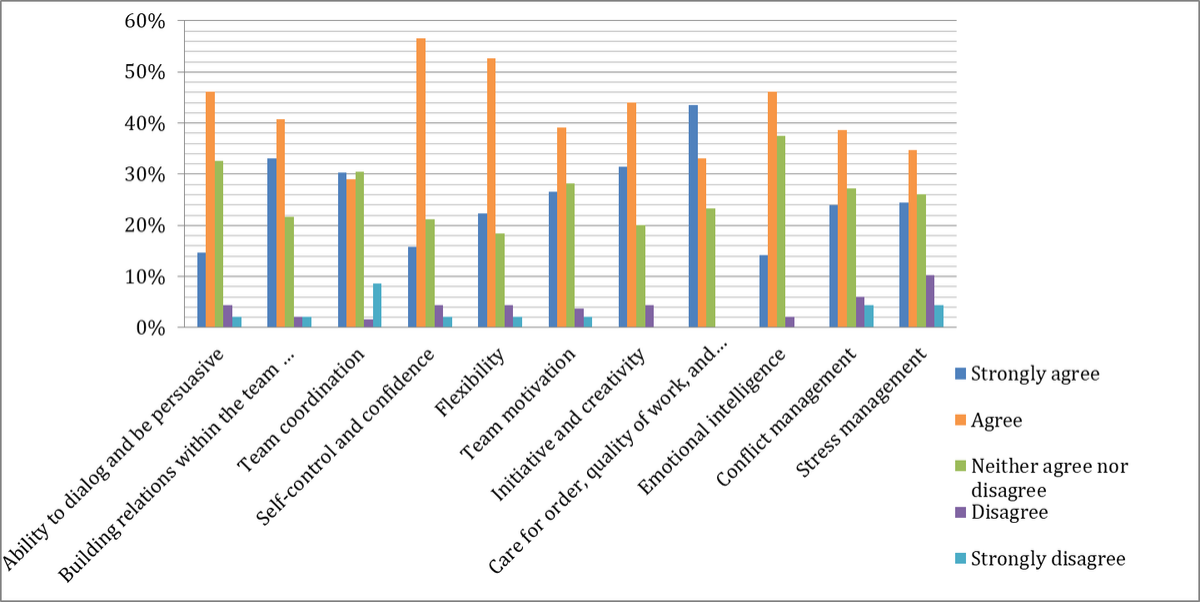
Improvement of social skills after MAEDRI game.

**Figure 4:**
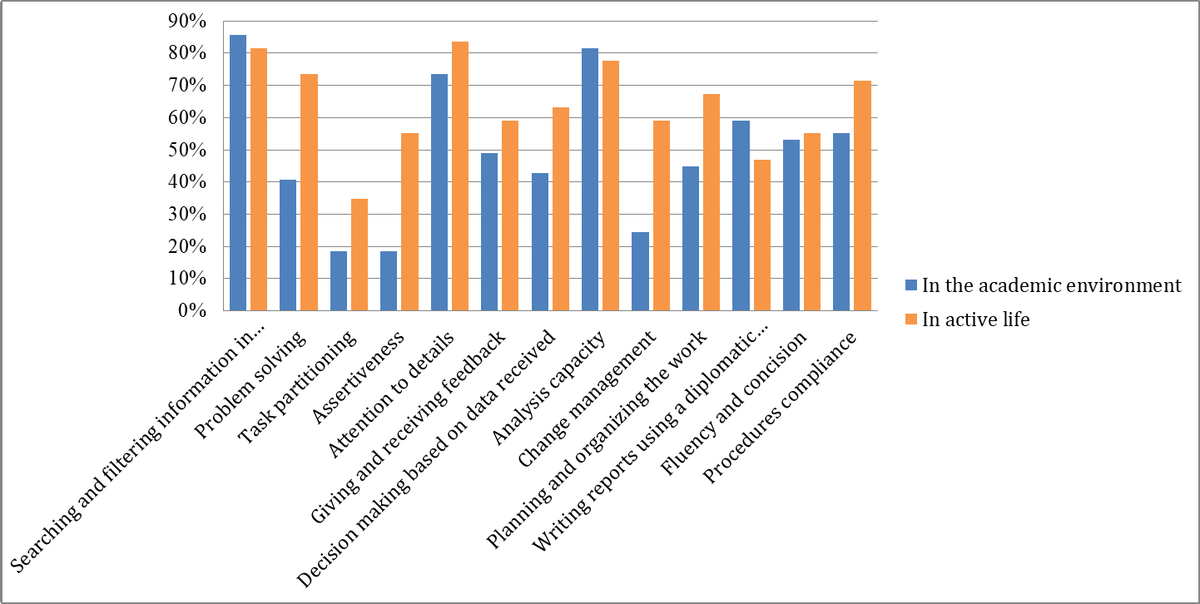
Usage of the acquired professional competencies in the academic environment and in active life.

Regarding social skills (Figure 3), we found the same tendency as with professional skills, although the approval rates were not as strong. During the game, several skills were emphasized and participants recognized that self-control, confidence, and flexibility were most substantially improved. Participants also agreed that the ability to dialog and be persuasive, development of emotional intelligence, care for order, attention to quality of work, and accuracy were all important skills that were developed by the simulation. In comparison with professional skills, 53% (26/49) of respondents thought that the exercise did not improve their social skills (or had only a minimal impact). This may be partly explained by the fact that, at least at the master’s level, students already have several years of working experience, and those skills may have already been developed in their working environments. In the second part of the questionnaire, respondents were asked if they used the specified competencies in the academic environment or in active life environment. In line with our hypotheses, our results show that the competencies developed by the game are important for preparing the students for the active life (Figure 4).

We found 3 exceptions where the respondents thought that those competencies have been slightly more useful in the academic environment than during their active life. These findings are obvious, and some competencies, such as searching and filtering information, writing reports using a diplomatic language, and analysis capacity, are used intensively during university studies. The same competencies are found useful in the active life only by the participants who are working in international relations–related workplaces.

**Figure 5:**
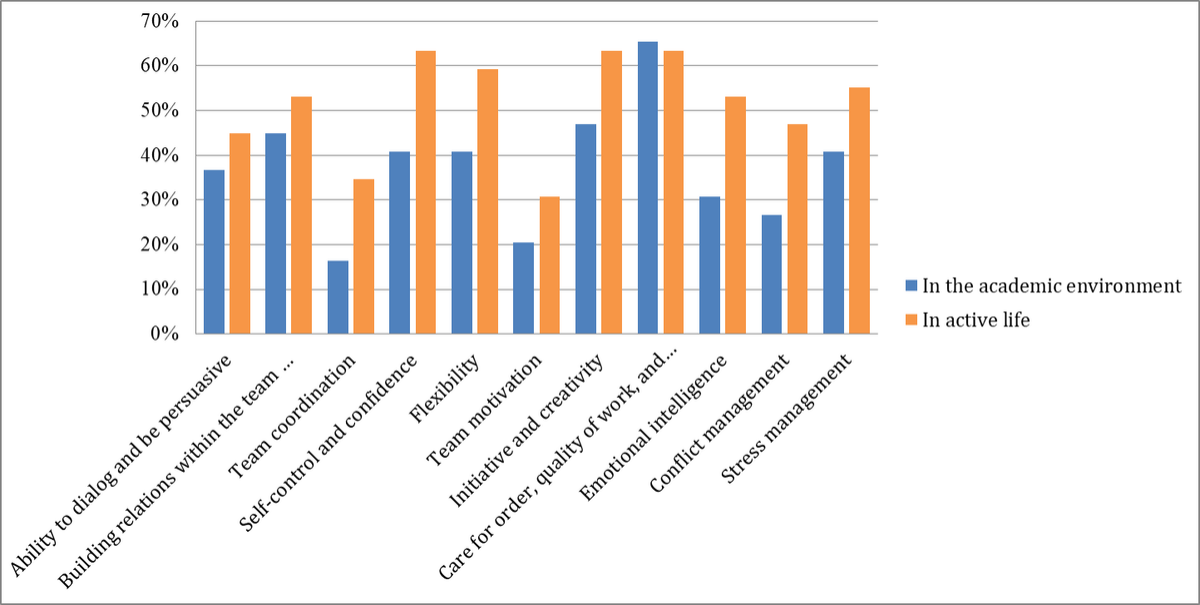
Usage of the acquired social skills in the academic environment and in the active life.

Regarding social skill usage, we found the same pattern (Figure 5). Although the respondents considered that those skills existed to a certain extent even before the simulation, an important part of respondents indicated that the usage of those competencies was more helpful during their active life. Further research must be performed to investigate this trend.

## Discussion

### Principal Findings

We developed the MAEDRI role play game and tested its impact on experiential learning in international relations and diplomacy studies. Our results reveal that MAEDRI can be easily implemented in the curriculum and can serve as an effective tool to boost knowledge and to form or enhance a series of competencies transferrable to the professional life. Our studies cover 4 years of annual simulations and were designed to analyze mainly the transfer of a number of professional competencies and social skills. In addition to confirming our underlying hypotheses, our results demonstrate that the overall effect of the learning game is in fact more complex and impacts, albeit at a lower extent, several social competencies. It was rewarding to observe that some of those achievements were used not only during but also after the instruction period, pointing to the game’s potentially long-lasting impact. It is remarkable that this conclusion is supported even by former students who ended up choosing a different career path and are currently not working in international relation–related fields.

Most participants appreciated the role play experience and provided positive feedback, pointing to their appreciation for opportunities to apply theoretical concepts into an experiential learning exercise, which closely mirrors the diplomatic real life.

The success of the MAEDRI simulation was observed in students’ motivation in maintaining a high level of day-to-day active participation in the exercise. In this respect, we observed good competition between different teams, striving to improve the postreach and postengagement rates of their reports, on the MAEDRI Facebook page. Our research is in line with the literature findings [29,43-45], where an increase in engagement by simulation was demonstrated in teaching political sciences. Moreover, for students who cannot participate in an internship, the exercise may generate equivalent competency acquisition.

The MAEDRI design offers a pragmatic pedagogical opportunity to demonstrate through experiential learning how diplomacy works in a day-to-day activity, as well as to engage in teamwork, enhance coordination within a hierarchical organization, increase work accuracy, and master time management. This is in line with how students are guided to discover knowledge through simulated experiences [46]. The MAEDRI role play simulation responds to the elements identified by Wilcox [46]: context (the students learn easier because they recognize the situation), practice (the exercise allows them to experiment in a safe manner with the theoretical concepts learned during their college studies), and experience (the students discover themselves what are the diplomatic day-to-day tasks). The experiential learning exercised during the MAEDRI role play focuses on developing or enhancing a number of capabilities, previously reported to be essential for political and social sciences [11,27-31].

Additionally, this data collection provides an insight into what activities are appreciated by the students and determines what could be done for improvement. The data from 4 consecutive game editions suggest that role play is a welcome alternative to the “classical” teaching approach often considered to be too theoretical by many Romanian students, especially those majoring in political sciences, international relations, or diplomacy.

The hierarchical organization that we created generates interactions between students within each compartment and between compartments in vertical hierarchies. We acknowledge that horizontal interactions between compartments situated on the same level were minimal or even null. This may be explained by the fact that the students are enrolled in different master’s programs and even when in the same program, they may be in different years of study. Additionally, for students who are not simultaneously enrolled in the same courses, interactions and collaborations outside the classwork may be more difficult to establish. This exercise confirmed that tendency, revealing that participants interacted almost exclusively within their group or with colleagues they were acquainted with. Next steps focus on improving the exercise by enhancing interactions between compartments of the same rank, to promote the team spirit and create a feeling of belonging to a wider community within our university.

This project was designed to evaluate the capacity of our newly designed MAEDRI role play game to enhance knowledge transfer and acquisition of key skills for graduate students preparing for a career in international relations and diplomacy. Although we used a nonprobabilistic sample, we consider that the results are relevant to validate the research hypotheses.

The students’ feedback stressed the fact that the exercise reached its main goal: to allow them to put in practice theoretical concepts. Moreover, it highlighted the role of this game exercise as a motivating factor, providing a better understanding of real diplomats’ tasks and offering a snapshot of real professional situations where both their hard and soft skills have been exposed and enhanced. Ultimately, we demonstrate that this role play exercise had a positive effect on knowledge transfer and enabled enhancement of several competencies.

### Limitations

We acknowledge several study limitations. Because of the small sample size, this is an exploratory study and further studies with increased sample size (and control arm including) are needed to validate these findings. The number of questionnaire respondents represents approximately 33% (49/150) of the total number of participants and our current focus is on identifying effective strategies to improve uptake.

We also note that the real impact of certain factors is difficult to assess; for example, those that might contribute to skill development in the workplace. Further research will try to evaluate the influence of such factors in the immediate as well as in long-term professional development.

Another limitation refers to a lack of control group. Further research will address this limitation.

### Conclusions

In summary, we have designed and implemented a new role play game and acquired data from 4 consecutive years of annual simulations. Our study demonstrates that the incorporation of role play is an effective experiential learning method that helps graduate students in master’s programs to better understand and use international relations and diplomacy concepts and enhance professional competencies. Most study participants provided positive feedback and the MAEDRI game was easy to implement. We acknowledge several limitations that must be considered when evaluating and interpreting the results. Further steps should be taken to investigate how the simulation exercise may enhance interactivity between different teams situated on the same level, and further stimulate the development of social skills.

## Appendix

Multimedia Appendix 1 MAEDRI simulation game preparation checklist.

Multimedia Appendix 2 MAEDRI Facebook page.

Multimedia Appendix 3 Table of correlations.

## Abbreviations

CHERRIES: Checklist for Reporting Results of Internet E-Surveys
MFA: Ministry of Foreign Affairs
NATO: North Atlantic Treaty Organization
SGs: simulation games
UN: United Nations
