# Supplementary material for: Gamification in Diplomacy Studies as an Effective Tool for Knowledge Transfer: Questionnaire Study
Source: JMIR Serious Games. 2022 Apr 25;10(2):e32996. doi: 10.2196/32996 (PMC9086880; doi:10.2196/32996)
Supplement: Multimedia Appendix 1 [file games_v10i2e32996_app1.doc]

| **Activity** | **Students in management roles** | **Students in Simulated diplomatic missions roles** | **Teacher** |
| --- | --- | --- | --- |
| Preparation | Advertising and promoting the simulation game among students in order to have a large selection base | Applying for the simulation game on a voluntary basis | Supervision and coordination |
|  | Selecting the students | Proposing several countries around the globe as simulated diplomatic mission based on their interests |  |
| Kickoff meeting | Creating the structure of MAEDRI organizations and setting up the communication system | Searching, filtering and reading the media in their „home” country. Selecting the most relevant sources which will be used during the simulation | Supervision and feedback |
|  | Taking over all credential for the MAEDRI Facebook page | Training sessions offered by professors and alumni | Supervision and feedback |
| Simulation | Coordinating their team  Reading and summarizing the reports.  Sending their daily feedback to the supervised teams  Publishing the selected reports on the MAEDRI Facebook page on a daily basis.  Giving and receiving feedback | Reading daily the selected Internet sources from the „simulated home country”. Drafting the report and sending it to the respective Simulated directorate. Sending feedback to the management team | Supervision |
| Debriefing | Collecting feedback from participants, analyzing data furnished by Facebook analytics and preparing the report for the exercise |  | Individual and group feedback |
