# Supplementary material for: Gamification in Diplomacy Studies as an Effective Tool for Knowledge Transfer: Questionnaire Study
Source: JMIR Serious Games. 2022 Apr 25;10(2):e32996. doi: 10.2196/32996 (PMC9086880; doi:10.2196/32996)
Supplement: Multimedia Appendix 2 [file games_v10i2e32996_app2.doc]

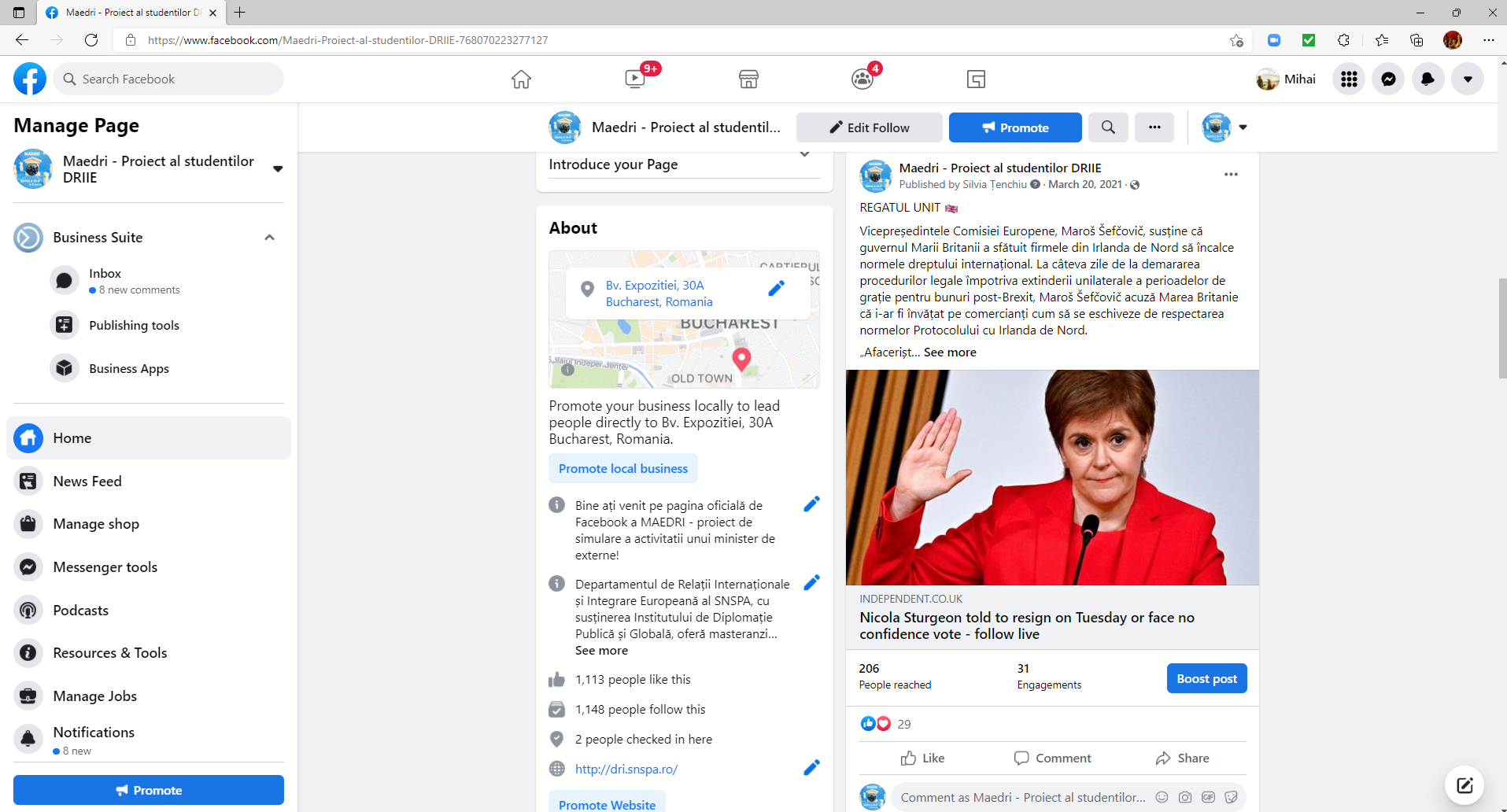


Prinscreen of the MAEDRI Facebook page [Accessed on Jan 20, 2022]

URL: https://www.facebook.com/pages/MAEDRI-Proiect-al-studentilor-DN/768070223277127?sk=info&tab=page_info
