# Supplementary material for: Gamification in Diplomacy Studies as an Effective Tool for Knowledge Transfer: Questionnaire Study
Source: JMIR Serious Games. 2022 Apr 25;10(2):e32996. doi: 10.2196/32996 (PMC9086880; doi:10.2196/32996)
Supplement: Multimedia Appendix 3 [file games_v10i2e32996_app3.doc]

| **Skills** | **A** | **B** | **C** | **D** | **E** | **F** | **G** | **H** | **I** | **J** | **K** | **L** | **M** | **N** | **O** | **P** | **Q** | **R** | **S** | **T** | **U** | **V** | **X** | **Y** |
| --- | --- | --- | --- | --- | --- | --- | --- | --- | --- | --- | --- | --- | --- | --- | --- | --- | --- | --- | --- | --- | --- | --- | --- | --- |
| **A** | 1.00 |  |  |  |  |  |  |  |  |  |  |  |  |  |  |  |  |  |  |  |  |  |  |  |
| **B** | 0.56 | 1.00 |  |  |  |  |  |  |  |  |  |  |  |  |  |  |  |  |  |  |  |  |  |  |
| **C** | 0.46 | 0.55 | 1.00 |  |  |  |  |  |  |  |  |  |  |  |  |  |  |  |  |  |  |  |  |  |
| **D** | 0.35 | 0.51 | 0.48 | 1.00 |  |  |  |  |  |  |  |  |  |  |  |  |  |  |  |  |  |  |  |  |
| **E** | 0.46 | 0.50 | 0.51 | 0.64 | 1.00 |  |  |  |  |  |  |  |  |  |  |  |  |  |  |  |  |  |  |  |
| **F** | 0.38 | 0.45 | 0.39 | 0.54 | 0.58 | 1.00 |  |  |  |  |  |  |  |  |  |  |  |  |  |  |  |  |  |  |
| **G** | 0.44 | 0.61 | 0.35 | 0.47 | 0.53 | 0.59 | 1.00 |  |  |  |  |  |  |  |  |  |  |  |  |  |  |  |  |  |
| **H** | 0.52 | 0.68 | 0.33 | 0.45 | 0.53 | 0.42 | 0.69 | 1.00 |  |  |  |  |  |  |  |  |  |  |  |  |  |  |  |  |
| **I** | 0.31 | 0.40 | 0.55 | 0.37 | 0.30 | 0.31 | 0.40 | 0.35 | 1.00 |  |  |  |  |  |  |  |  |  |  |  |  |  |  |  |
| **J** | 0.63 | 0.47 | 0.60 | 0.43 | 0.34 | 0.27 | 0.28 | 0.37 | 0.46 | 1.00 |  |  |  |  |  |  |  |  |  |  |  |  |  |  |
| **K** | 0.56 | 0.56 | 0.37 | 0.31 | 0.38 | 0.32 | 0.42 | 0.51 | 0.36 | 0.56 | 1.00 |  |  |  |  |  |  |  |  |  |  |  |  |  |
| **L** | 0.35 | 0.32 | 0.35 | 0.28 | 0.54 | 0.32 | 0.38 | 0.49 | 0.34 | 0.33 | 0.53 | 1.00 |  |  |  |  |  |  |  |  |  |  |  |  |
| **M** | 0.47 | 0.39 | 0.57 | 0.30 | 0.47 | 0.21 | 0.26 | 0.42 | 0.31 | 0.69 | 0.56 | 0.40 | 1.00 |  |  |  |  |  |  |  |  |  |  |  |
| **N** | 0.49 | 0.43 | 0.56 | 0.44 | 0.44 | 0.54 | 0.47 | 0.38 | 0.58 | 0.43 | 0.41 | 0.35 | 0.35 | 1.00 |  |  |  |  |  |  |  |  |  |  |
| **O** | 0.40 | 0.44 | 0.37 | 0.38 | 0.53 | 0.53 | 0.43 | 0.49 | 0.27 | 0.33 | 0.51 | 0.38 | 0.31 | 0.70 | 1.00 |  |  |  |  |  |  |  |  |  |
| **P** | 0.35 | 0.29 | 0.35 | 0.22 | 0.21 | 0.30 | 0.36 | 0.25 | 0.35 | 0.23 | 0.14 | 0.19 | 0.23 | 0.48 | 0.33 | 1.00 |  |  |  |  |  |  |  |  |
| **Q** | 0.44 | 0.52 | 0.53 | 0.27 | 0.38 | 0.39 | 0.43 | 0.53 | 0.40 | 0.44 | 0.52 | 0.47 | 0.50 | 0.42 | 0.45 | 0.30 | 1.00 |  |  |  |  |  |  |  |
| **R** | 0.39 | 0.43 | 0.34 | 0.46 | 0.44 | 0.39 | 0.42 | 0.46 | 0.47 | 0.44 | 0.55 | 0.42 | 0.37 | 0.37 | 0.43 | 0.08 | 0.55 | 1.00 |  |  |  |  |  |  |
| **S** | 0.31 | 0.48 | 0.63 | 0.51 | 0.50 | 0.45 | 0.41 | 0.45 | 0.45 | 0.59 | 0.35 | 0.41 | 0.42 | 0.54 | 0.50 | 0.46 | 0.50 | 0.26 | 1.00 |  |  |  |  |  |
| **T** | 0.40 | 0.48 | 0.29 | 0.39 | 0.49 | 0.41 | 0.32 | 0.53 | 0.25 | 0.49 | 0.55 | 0.48 | 0.55 | 0.24 | 0.39 | 0.16 | 0.58 | 0.64 | 0.41 | 1.00 |  |  |  |  |
| **U** | 0.54 | 0.42 | 0.36 | 0.45 | 0.61 | 0.35 | 0.35 | 0.52 | 0.15 | 0.59 | 0.52 | 0.55 | 0.59 | 0.39 | 0.43 | 0.23 | 0.41 | 0.39 | 0.52 | 0.69 | 1.00 |  |  |  |
| **V** | 0.37 | 0.36 | 0.19 | 0.37 | 0.28 | 0.12 | 0.36 | 0.44 | 0.43 | 0.42 | 0.20 | 0.29 | 0.28 | 0.31 | 0.17 | 0.27 | 0.33 | 0.35 | 0.37 | 0.39 | 0.39 | 1.00 |  |  |
| **X** | 0.41 | 0.61 | 0.41 | 0.47 | 0.29 | 0.43 | 0.58 | 0.55 | 0.54 | 0.38 | 0.37 | 0.18 | 0.22 | 0.42 | 0.30 | 0.42 | 0.47 | 0.48 | 0.54 | 0.37 | 0.17 | 0.54 | 1.00 |  |
| **Y** | 0.38 | 0.64 | 0.39 | 0.53 | 0.39 | 0.35 | 0.57 | 0.59 | 0.44 | 0.47 | 0.46 | 0.24 | 0.37 | 0.33 | 0.37 | 0.24 | 0.50 | 0.62 | 0.48 | 0.56 | 0.35 | 0.59 | 0.86 | 1.00 |

Legend:

A - Searching and filtering information in virtual space; B - Problem solving; C - Task partitioning; D – Assertiveness; E - Attention to details; F - Giving and receiving feedback; G - Decision making based on data received; H - Analysis capacity; I - Change management; J - Planning and organizing the work; K - Writing reports using a diplomatic language; L - Fluency and concision; M - Procedures compliance; N - Ability to dialog and be persuasive; O - Building relations within the team; P - Team coordination; Q – Self-control and confidence; R – Flexibility; S - Team motivation; T - Initiative and creativity; U -Care for order, quality of work, and accuracy; V -Emotional intelligence; X - Conflict management; Y - Stress management
